# Tight upper bounds for semi-online scheduling on two uniform machines with known optimum

**DOI:** 10.1007/s10100-017-0481-z

**Published:** 2017-06-14

**Authors:** György Dósa, Armin Fügenschuh, Zhiyi Tan, Zsolt Tuza, Krzysztof Węsek

**Affiliations:** 1 Department of Mathematics, University of Pannonia, Veszprém, Hungary; 2 Helmut Schmidt University/University of the Federal Armed Forces Hamburg, Holstenhofweg 85, 22043 Hamburg, Germany; 3 Department of Mathematics, Zhejiang University, Hangzhou, People’s Republic of China; 4 Department of Computer Science and Systems Technology, University of Pannonia, Veszprém, Hungary; 5 Alfréd Rényi Institute of Mathematics, Hungarian Academy of Sciences, Budapest, Hungary; 6 Faculty of Mathematics and Information Science, Warsaw University of Technology, ul. Koszykowa 75, 00-662 Warsaw, Poland

**Keywords:** Scheduling, Semi-online algorithm, Makespan minimization, Mixed-integer linear programming

## Abstract

We consider a semi-online version of the problem of scheduling a sequence of jobs of different lengths on two uniform machines with given speeds 1 and *s*. Jobs are revealed one by one (the assignment of a job has to be done before the next job is revealed), and the objective is to minimize the makespan. In the considered variant the optimal offline makespan is known in advance. The most studied question for this online-type problem is to determine the optimal competitive ratio, that is, the worst-case ratio of the solution given by an algorithm in comparison to the optimal offline solution. In this paper, we make a further step towards completing the answer to this question by determining the optimal competitive ratio for *s* between $\frac{5 + \sqrt{241}}{12} \approx 1.7103$ and $\sqrt{3} \approx 1.7321$, one of the intervals that were still open. Namely, we present and analyze a compound algorithm achieving the previously known lower bounds.

## Introduction

Combinatorial optimization problems come with various paradigms on how an instance is revealed to a solving algorithm. The very common *offline* paradigm assumes that the entire instance is known in advance. On the opposite end, one can deal with the pure *online* scheme, where the instance is revealed part by part, unpredictable to the algorithm, and no further knowledge on these parts is assumed. In between these two extremes, and also highly relevant for many practical applications, are *semi-online* paradigms, where at least some characteristics of the instance in general are assumed to be known, for example, the total instance size or distributions of some internal values.

As a continuation of our work (Dósa et al. 2015a), we consider a semi-online variant of a scheduling problem for two uniform machines, that is defined as follows. Suppose that two machines M1 and M2 are processing a sequence of incoming jobs of varying lengths. Machine M1 has a speed of 1, so that a job of length $\ell$ is processed within $\ell$ units of time, whereas machine M2 has a speed of $s \ge 1$, so that a job of length $\ell$ can be processed within $\frac{\ell }{s}$ units of time. The load of a machine is the total size of jobs assigned to that machine (without dividing by the speed of the machine). This definition is non-standard, but in this way some of our calculations become simpler. The jobs must be assigned to the machines in an online fashion, so that the next job becomes visible only when the previous job has already been assigned. The goal is to find a schedule that minimizes the total makespan, that is, the point in time when the last job on either machine is finished. We assume that the optimal value of the makespan for the corresponding offline problem (where all jobs are known in advance), denoted by OPT is available to the scheduler, and can be taken into account during its assignment decisions.

We are interested in constructing an algorithm $\mathcal {A}$ that solves this semi-online problem, and achieves a small makespan. Of course, for a given instance *I* of the problem, the (offline) $\text {OPT} = \text {OPT} (I)$ value is a lower bound for the semi-online problem. Thus, we consider the competitive ratio $\frac{M_{\mathcal {A}}(I)}{\text {OPT} (I)} \ge 1$, where $M_{\mathcal {A}}(I)$ is the makespan value achieved by algorithm $\mathcal {A}$ when applied to instance *I*, as a performance measure.

The competitive ratio $r_{\mathcal {A}}$ of an algorithm $\mathcal {A}$ is then defined as the worst case of this ratio, that is, the supremum over all possible problem instances:

$$\begin{aligned} r_{\mathcal {A}} = \sup \left\{ \frac{M_{\mathcal {A}}(I)}{\text {OPT} (I)} : I \text{ is } \text{ an } \text{ instance }\right\} . \end{aligned}$$

One can try to bound the value of *r* from below by estimating the infimum of $r_{\mathcal {A}}$ over all algorithms $\mathcal {A}$, that is,

$$\begin{aligned} r^* := \inf \{r_{\mathcal {A}} : \mathcal {A} \text{ an } \text{ algorithm }\}. \end{aligned}$$

We call $r^*$ the optimal competitive ratio. An algorithm $\mathcal {A}$ is said to be *r*-competitive, if for any instance *I* its performance is bounded by *r* from above: $\frac{M_{\mathcal {A}}(I)}{\text {OPT} (I)} \le r$. An optimal algorithm in this sense is $r^*$-competitive.

### Survey of the literature

The problem of scheduling a set of jobs on *m* (possibly not identical) machines with the objective to minimize the makespan (maximum completion time), with the jobs being revealed one-by-one, is a classic online algorithmic problem. Starting with results of Graham (1969), much work has been done in this field (see for example Albers 1999; Berman et al. 2000; Ebenlendr and Sgall 2007; Faigle et al. 1989; Fleischer and Wahl 2000; Gormley et al. 2000), although even if we restrict only to the case of identical machines, the optimal ratio is still not known in general.

From both the theoretical and practical point of view, it may be important to investigate semi-online models, in which some additional information or relaxation is available. In this work we consider the scheme in which only the optimal offline value is known in advance (OPT version); however it is worth mentioning a strong relation with another semi-online version of the described scheduling problem, in which only the sum of jobs is known (SUM version) (Angelelli et al. 2004, 2007, 2008; Dósa et al. 2011; Kellerer et al. 1997; Lee and Lim 2013; Ng et al. 2009). Namely, for a given number *m* of uniform (possibly non-identical) machines the optimal competitive ratio for the OPT version is at most the competitive ratio of the SUM version [see Dósa et al. (2011); for equal speeds this was first implicitly stated by Cheng et al. (2005)].

For a more detailed overview of the literature on online and various semi-online variants, we refer to the survey of Tan and Zhang (2013).

Azar and Regev (2001) introduced the OPT version on (two or more) identical machines under the name of bin stretching, and this case was studied further by Cheng et al. (2005) and by Lee and Lim (2013). However, knowing the relation between the OPT and SUM versions, the first upper bound for two equal-speed machines follows from the work of Kellerer et al. (1997) on the SUM version.

We must mention some recent papers in the case of identical machines by Gabay et al. (2015) and Böhm et al. (2016a, b). The main reason is the similarity of attitudes by which we and those authors approach the problems: they also use separate algorithms for certain good situations. In particular, Böhm et al. (2016b) makes this method very explicit. During the execution of some (online) algorithm, we sometimes meet some “good situations”. This means that the schedule can surely be finished without any bigger problem or surprise, i.e. keeping the targeted worst-case ratio. And the more difficult cases are handled by some other algorithm which is exactly trained to deal with the difficult situations. We do this idea by handling the good situations by algorithm FinalCases, and the remaining not so friendly cases by another algorithm, called InitialCases. The separation of the final and other cases seems to be very natural for this type of problem.

In this work we are interested in the OPT version on two uniform machines with non-identical speeds, therefore we summarize previous results for this case. Recall that speeds of machines are 1 and *s*. Known bounds on the optimal competitive ratio $r^{*}$ are expressed in terms of *s*.

Studies on this version of the problem were initiated by Epstein (2003). She provided the following bounds:

$$\begin{aligned} r^{*}(s) : \left\{ \begin{array}{l@{\quad }l} r^{*}(s)\in \Big [ \frac{3s+1}{3s}, \frac{2s + 2}{2s + 1}\Big ] &{}\hbox { for }s\in [1, q_{E} \approx 1.1243]\\ r^{*}(s)\in \Big [s\big (\frac{3}{4}+\frac{\sqrt{65}}{20}\big ), \frac{2s + 2}{2s + 1}\Big ] &{}\hbox { for }s\in \big [q_{E}, \frac{1 + \sqrt{65}}{8} \approx 1.1328\big ]\\ r^{*}(s)=\frac{2s+2}{2s+1} &{}\hbox { for }s\in \big [\frac{1 + \sqrt{65}}{8} , \frac{1 + \sqrt{17}}{4} \approx 1.2808 \big ]\\ r^{*}(s)=s &{}\hbox { for }s\in \big [ \frac{1 + \sqrt{17}}{4} , \frac{1 + \sqrt{3}}{2} \approx 1.3660 \big ]\\ r^{*}(s)\in \Big [ \frac{2s+1}{2s}, s \Big ] &{}\hbox { for }s\in \big [\frac{1 + \sqrt{3}}{2}, \sqrt{2} \approx 1.4142\big ]\\ r^{*}(s)\in \Big [ \frac{2s+1}{2s}, \frac{s+2}{s+1} \Big ] &{}\hbox { for } s\in \big [ \sqrt{2}, \frac{1 + \sqrt{5}}{2} \approx 1.6180 \big ]\\ r^{*}(s)\in \Big [ \frac{s+1}{2}, \frac{s+2}{s+1} \Big ] &{}\hbox { for }s\in \big [ \frac{1 + \sqrt{5}}{2}, \sqrt{3} \approx 1.7321 \big ]\\ r^{*}(s) = \frac{s+2}{s+1} &{}\hbox { for }s \ge \sqrt{3} \end{array} \ \right. \end{aligned}$$

where $q_{E}$ is the solution of $36x^{4} - 135x^{3} +45x^{2} +60x + 10 = 0$.

Ng et al. (2009) studied this problem with comparison to the SUM version. They presented algorithms giving the upper bounds

$$\begin{aligned} r^{*}(s) \le \left\{ \begin{array}{l@{\quad }l} \frac{2s+1}{2s} &{}\hbox { for }s\in \big [ \frac{1 + \sqrt{3}}{2}, \frac{1 + \sqrt{21}}{4} \approx 1.3956 \big ]\\ \frac{6s+6}{4s+5} &{}\hbox { for }s\in \big [ \frac{1 + \sqrt{21}}{4}, \frac{1 + \sqrt{13}}{3} \approx 1.5352 \big ]\\ \frac{12s+10}{9s+7} &{}\hbox { for }s\in \big [ \frac{1 + \sqrt{13}}{3}, \frac{5 + \sqrt{241}}{12} \approx 1.7103 \big ]\\ \frac{2s+3}{s+3} &{}\hbox { for }s\in \big [ \frac{5 + \sqrt{241}}{12}, \sqrt{3} \big ] \end{array} \ \right. \end{aligned}$$

and proved the following lower bounds:

$$\begin{aligned} r^{*}(s) \ge \left\{ \begin{array}{l@{\quad }l} \frac{3s+5}{2s+4} &{}\hbox { for }s\in \big [ \sqrt{2}, \frac{\sqrt{21}}{3} \approx 1.5275 \big ]\\ \frac{3s+3}{3s+1} &{}\hbox { for }s\in \big [ \frac{\sqrt{21}}{3}, \frac{5 + \sqrt{193}}{12} \approx 1.5744 \big ]\\ \frac{4s+2}{2s+3} &{}\hbox { for }s\in \big [ \frac{5 + \sqrt{193}}{12}, \frac{7 + \sqrt{145}}{12} \approx 1.5868 \big ]\\ \frac{5s+2}{4s+1} &{}\hbox { for }s\in \big [ \frac{7 + \sqrt{145}}{19}, \frac{9 + \sqrt{193}}{14} \approx 1.6352 \big ]\\ \frac{7s+4}{7s} &{}\hbox { for }s\in \big [ \frac{9 + \sqrt{193}}{14}, \frac{5}{3} \big ]\\ \frac{7s+4}{4s+5} &{}\hbox { for }s\in \big [ \frac{5}{3}, \frac{5 + \sqrt{73}}{8} \approx 1.6930 \big ] \end{array} \right. \end{aligned}$$

Dósa et al. (2011) considered this version together with the SUM version. Their results included the bounds

$$\begin{aligned} r^{*}(s)\ge & {} \left\{ \begin{array}{l@{\quad }l} \frac{8s+5}{5s+5} &{}\hbox { for }s\in \big [ \frac{5 + \sqrt{205}}{18}, \frac{1 + \sqrt{31}}{6} \approx 1.0946 \big ]\\ \frac{2s+2}{2s+1} &{}\hbox { for }s\in \big [ \frac{1 + \sqrt{31}}{6}, \frac{1 + \sqrt{17}}{4} \approx 1.2808 \big ] \end{array} \ \right. \\ r^{*}(s)\le & {} \left\{ \begin{array}{l@{\quad }l} \frac{3s+1}{3s} &{}\hbox { for }s\in \big [ 1, q_{D} \approx 1.071 \big ]\\ \frac{7s+6}{4s+6} &{}\hbox { for }s\in \big [ q_{D} , \frac{1 + \sqrt{145}}{12} \approx 1.0868 \big ] \end{array} \ \right. \end{aligned}$$

where $q_{D}$ is the unique root of the equation $3s^{2}(9s^{2} -s-5)=(3s+1)(5s+5-6s^{2})$.

Finally, the recent manuscript (Dósa et al. 2015a) whose results complement this work of the present authors provided the following lower bounds:

$$\begin{aligned} r^*(s)\ge \left\{ \begin{array}{l@{\quad }l} \frac{6s+6}{4s+5} &{} \text {for } s\in \big [\frac{\sqrt{21}+1}{4} \approx 1.3956 , \frac{\sqrt{73}+3}{8} \approx 1.443 \big ] \\ \frac{12s+10}{9s+7} &{}\hbox { for }s\in \big [ \frac{5}{3}, \frac{13+\sqrt{1429}}{30} \approx 1.6934 \big ] \\ \frac{18s+16}{16s+7}, &{}\hbox { for }s\in \big [ \frac{13+\sqrt{1429}}{30}, \frac{30+7\sqrt{186}}{74} \approx 1.6955 \big ] \\ \frac{8s+7}{3s+10}, &{}\hbox { for }s\in \big [ \frac{30+7\sqrt{186}}{74} , \frac{31+\sqrt{8305}}{72}\approx 1.6963 \big ] \\ \frac{12s+10}{9s+7} &{}\hbox { for }s\in \big [ \frac{31+\sqrt{8305}}{72}, \frac{4+\sqrt{133}}{9} \approx 1.7258 \big ] \end{array} \ \right. \end{aligned}$$

Here we collected only a brief summary of known bounds; for further details about previous results we refer to Dósa et al. (2015a).

### Our contribution

After the work of Dósa et al. (2015a), between $\frac{5}{3}$ and $\sqrt{3}$ there are two intervals, namely $\big [\frac{13+\sqrt{1429}}{30}, \frac{31+\sqrt{8305}}{72} \big ] \approx \big [ 1.6934, 1.6963 \big ]$ that we call *narrow interval* and $[\frac{5+\sqrt{241}}{12}, \sqrt{3}] \approx [1.7103, 1.7321]$ that we call *wide interval*, where the question remained open regarding the tight value of the competitive ratio.

In the narrow interval the upper bound is very close to the lower bound (the biggest gap is still smaller than 0.000209), so in this paper we focus on the wide interval, for which we present an optimal compound algorithm which has a competitive ratio that equals the previously known lower bounds.

We apply the method of “safe sets”. This idea probably first applied in Epstein (2003). The concept is used also later by Ng et al. (2009) and Angelelli et al. (2010) (called “green set” in the latter), and also used by Dósa et al. (2011). Once those sets are properly defined (cf. Fig. 2), we try to assign the next job in the sequence to a machine where its completion time will be in some safe set. In case of the quoted papers, the safe sets are defined in such a way that the next property holds in *any* case: after some initial phase when the loads of both machines are low, a job will surely arrive that can be assigned into a safe set. In other words, the boundaries of the safe sets are *optimized* in the way that the best possible competitive ratio would be reached while the above property holds.

Now, we make a crucial modification extending the power of the method. We realize that, keeping the above property, the algorithm *cannot be optimal* in the considered interval of speeds, therefore we do not insist on this property for defining the boundaries of the safe sets. We are less restrictive as we allow the possibility that during the scheduling process, some relatively big job may arrive, which cannot be assigned within a safe set. But it turns out that this unpleasant case can be handled by another kind of algorithm. So, for any incoming job first we try our algorithm “Final Cases” which uses the safe sets, to assign the actual job into a safe set if possible. If this is not possible, we apply our second algorithm “Initial Cases” to assign the job.

**Fig. 1:**
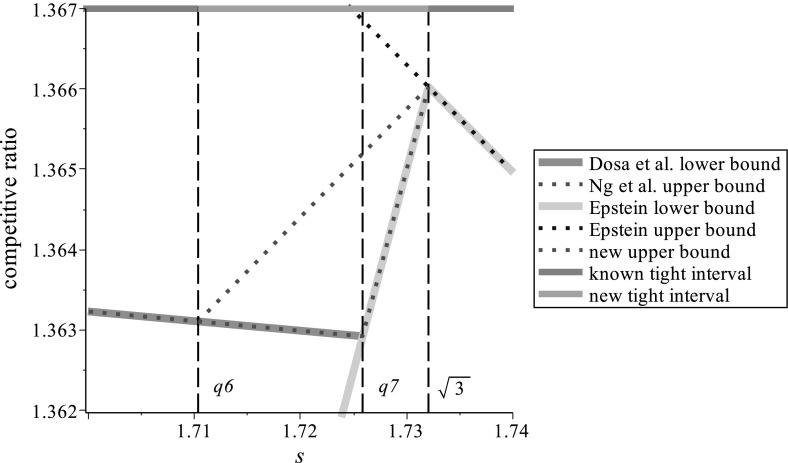
Known and new upper and lower bounds from Epstein (2003), Ng et al. (2009), and Dósa et al. (2015a)

We further show that our algorithm matches the best known algorithm of Ng et al. (2009) regarding the competitive ratio on the interval $[\frac{1+\sqrt{13}}{3},\frac{5+\sqrt{241}}{12}] \approx [1.5352, 1.7103]$. For a visual comparison of the previously known results and our contribution we refer to Fig. 1. Whenever the dotted line (that represents an upper bound) is on an unbroken line (that represents a lower bound), the optimal competitive ratio is known.

## Notions and definitions

Let $q_0:=\frac{1+\sqrt{13}}{3} \approx 1.5352$, which is the positive solution of $\frac{6s+6}{4s+5}=\frac{12s+10}{9s+7}$.

Let $q_{6} :=\frac{5 + \sqrt{241}}{12} \approx 1.7103$, which is the positive solution of $\frac{12s+10}{9s+7}=\frac{2s+3}{s+3}$.

Let $q_{7} := \frac{4+\sqrt{133}}{9} \approx 1.7258$, which is the positive solution of $\frac{12s+10}{9s+7}=\frac{s+1}{2}$.

**Fig. 2:**
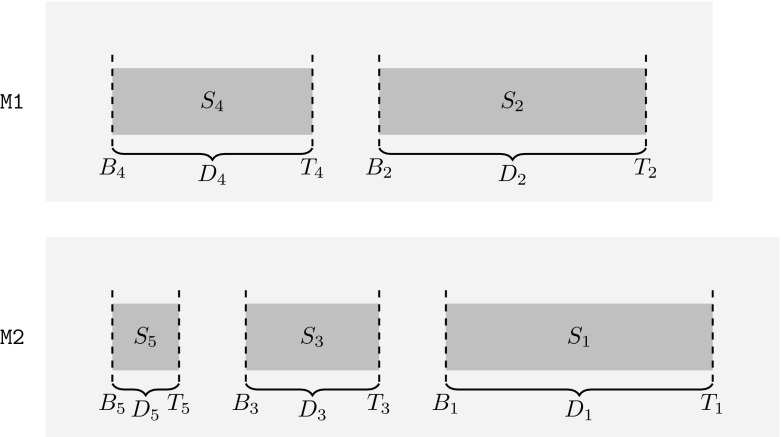
Safe sets

We note that the values $q_6$ and $q_7$ were already defined in the paper (Dósa et al. 2015a). Then the wide interval is $[q_6,\sqrt{3}]$. For the remainder of this article we consider values of *s* from the wide interval only. We define

$$\begin{aligned} r(s):=\left\{ \begin{array}{l@{\quad }l@{\quad }l} r_{2}(s):=\frac{12s+10}{9s+7}, &{}\hbox { if }q_{6}\le s\le q_{7}\approx 1.7258, &{}\hbox { i.e.},\ s\hbox { is regular,}\\ r_5(s):=\frac{s+1}{2}, &{}\hbox { if }q_{7}\le s \le \sqrt{3}, &{}\hbox { i.e.},\ s\hbox { is large}. \end{array} \ \right. \end{aligned}$$

We remark that the value $r_2(s)$ is the same as in our preceding paper (Dósa et al. 2015a). The speeds to the left from the narrow interval (which are not considered in this paper) were called smaller regular speeds. The speeds to the right of the narrow interval were called bigger regular speeds, now we call these speeds simply as regular. The value $r_5(s)$ is Epstein’s lower bound from Epstein (2003) on the right side of the wide interval. Note also that the graph of $r_2(s)$ can be seen on the figure between $q_6$ and $q_7$, where the dotted line touches the unbroken line. Similarly, the graph of $r_5(s)$ appears between $q_7$ and $\sqrt{3}$, where the dotted line touches the unbroken line.

Let $\text {OPT}$ and $\text {SUM}$ mean, respectively, the known optimum value, and the total size of the jobs. Note that $\text {SUM} \le (s+1)\cdot OPT$, and the size of any job is at most $s\cdot OPT$. We denote the prescribed competitive ratio (that we do not want to violate) by *r*.

The optimum value is assumed to be known, and for sake of simplicity we will assume that OPT is equal to 1. (This can be assumed without loss of generality by normalization, i.e., dividing all of the job lengths by the optimal makespan.) Then we define five safe sets $S_i := [B_i, T_i]$ with size $D_i := T_i - B_i$ for $i = 1,\ldots ,5$ as follows (see also Fig. 2):

1. $$B_{1}:=s+1-r$$ and $$T_{1}:=rs$$, thus $$D_{1}=( s+1) ( r-1) $$,
2. $$B_{2}:=s+1-sr$$ and $$T_{2}:=r$$, thus $$D_{2}=( s+1) ( r-1) $$,
3. $$B_{3}:=2s-2r-rs+2$$ and $$T_{3}:=s(r-1)$$, thus $$D_{3}=2r-3s+2rs-2$$,
4. $$B_{4}:=4s-2r-3rs+3$$ and $$T_{4}:=r-1$$, thus $$D_{4}=( 3r-4) (s+1) $$,
5. $$B_{5}:=6s-5r-4rs+6$$ and $$T_{5}:=10s-7r-7rs+9$$, thus $$D_{5}=4s-2r-3rs+3$$.

These sets define time intervals, and they are called “safe” because if the load of the machine is in this interval, this enables a “smart” algorithm (as the one we introduce later) to finish the schedule by not violating the desired competitive ratio. In other words, from the point of view of an algorithm (which wishes to keep the competitive ratio low), we want to assign the actual job in a way that the increased load of some machine will be inside a safe set.

## Properties

In this section we summarize some technical properties and estimations of the definitions and notions from the previous section, which are needed within the computations in the subsequent sections.

### Lemma 1

$$r_5(s) \ge r_2(s)$$ for $$s \ge q_{7}$$.

### Proof

$r_5(s) - r_2(s) = \frac{s+1}{2} - \frac{12s+10}{9s+7} = \frac{9s^2 - 8s - 13}{2 (9s + 7)} \ge 0$, which is true since $9s^2 - 8s - 13 \ge 0$ holds if and only if $s \le \frac{4-\sqrt{133}}{9}$ or $s \ge \frac{4+\sqrt{133}}{9} = q_{7}$. $\square$

### Lemma 2

The following inequalities hold in the entire considered domain of the function *r*, i.e., for all $s \in [q_6,\sqrt{3}]$.

1. $$\frac{3s+2}{2s+2}< \frac{4}{3}< 1.35< r(s)< \min \left\{ \frac{4s+3}{3s+2}, \frac{s+2}{s+1}\right\}< \frac{2s+1}{s+1} < 2$$.
2. $$ \frac{8s+7}{6s+5} \le r(s)$$.
3. $$\frac{s+3}{s+2}< \frac{7s+5}{5s+4}< \frac{s+1}{2} \le r(s) < \frac{6s+6}{4s+5}$$.

### Proof

The rightmost part in Lemma 2.1, i.e. $\frac{2s+1}{s+1} < 2$, holds trivially. All other claims in 2.1 and 2.2 but the ones which regard $r_5(s)$ are already proven in Dósa et al. (2015a), thus we give only this unproved part here. Moreover, we give the proof for 2.3, whose claims were not considered before.

1. The leftmost lower bound holds as $\frac{3s+2}{2s+2}< \frac{4}{3}$ is equivalent to $4(2s+2)-3(3s+2) = 2-s > 0$, and hence to $s < 2$. Further, it is easy to see that $r(s)=\frac{s+1}{2} > 1.35$, since $s>1.7$ in the domain of $r_5$. Regarding the upper bound, $\frac{2s+1}{s+1} > \frac{s+2}{s+1}$ holds trivially since $s>1$, thus it remains to show that $r < \min \left\{ \frac{s+2}{s+1},\frac{4s+3}{3s+2}\right\}$. Note that $\frac{4s+3}{3s+2}\ge \frac{s+2}{s+1}$ for positive *s* holds if and only if $(4s+3)(s+1)-(s+2)(3s+2)=s^{2}-s-1\ge 0$, i.e., $s \ge \frac{1+\sqrt{5}}{2} \approx 1.618$. Therefore, for large *s* we need to show only that $r < \frac{s+2}{s+1}$. We have $\frac{s+1}{2} - \frac{s+2}{s+1} < 0$, which holds since $s^2-3 \le 0$ is true.
2. For large *s* we get that $\frac{s+1}{2}\ge \frac{8s+7}{6s+5}$ holds if and only if $(6s+5)(s+1)-2(8s+7)=6s^{2}-5s-9\ge 0$, i.e., $s\ge \frac{5 + \sqrt{241}}{12} \approx 1.7103=q_6$ which is valid.
3. Regarding the leftmost inequality, $\frac{7s+5}{5s+4}-\frac{s+3}{s+2}=\frac{2\left( s-1\right) (s+1)}{\left( s+2\right) \left( 5s+4\right) }>0$ trivially holds. The next inequality holds since $\frac{s+1}{2}- \frac{7s+5}{5s+4}=\frac{5s^{2}-5s-6}{2(5s+4)} >0$ holds if $s > \frac{5+\sqrt{145}}{10} \approx 1.7042$ (and this value is smaller than $q_6$). Regarding $r(s) \ge \frac{s+1}{2}$, for large speeds the inequality holds trivially (with equality) and for regular speeds we have already seen the validity of the inequality in Lemma  1. Thus we are done with the lower bound; let us see the upper bound. For regular *s* we have $\frac{6s+6}{4s+5} - \frac{12s+10}{9s+7} = \frac{6s^2-4s-8}{(9s+7)(4s+5)} \ge 0$, which is true, since $6s^2-4s-8 \ge 0$ for $s \le \frac{1-\sqrt{13}}{3}$ and $s \ge \frac{1+\sqrt{13}}{3} = q_0 \approx 1.535$. For large *s* we have $\frac{6s + 6}{4s + 5} - \frac{s + 1}{2} = \frac{-4s^2 + 3s + 7}{2(4s+5)} = \frac{(s + 1)(7 - 4s)}{2(4s+5)} \ge 0$, which is true since $s \le 1.75$.

$$\square $$

In the next lemma we state some properties of the safe sets. Note that an alternative option to define the safe sets would be to require these properties below.

### Lemma 3

1. $$D_{1}=D_{2}$$,
2. $$T_{1}-T_{3}=s$$ and $$T_{2}-T_{4}=1$$,
3. $$B_{3}=B_{1}-D_{1}$$,
4. $$B_{4}=B_{2}-D_{3}$$,
5. $$B_{5}=B_{3}-D_{4}$$,
6. $$T_{5}=B_{5}+B_{4}$$.

### Proof

Proofs of the equalities in Lemmas 3.1–3.4 were given in Dósa et al. (2015a). Since these proofs use nothing else than the definition of the safe sets, we do not repeat them. For proving 3.5 and 3.6 we use again the definitions of the boundaries.

5. $$\begin{aligned} B_5 + D_4= & {} (6s-5r-4rs+6) + (3r-4) (s+1) \\= & {} 2s-2r-rs+2 = B_3. \end{aligned}$$
6. $$\begin{aligned} B_5 + B_4= & {} (6s-5r-4rs+6) + (4s-2r-3rs+3) \\= & {} 10s-7r-7rs+9 = T_5. \end{aligned}$$

$$\square $$

The next lemma proves that the safe sets are well defined in the sense that they are disjoint sets, and follow each other in the described order on the machines.

### Lemma 4

The following inequalities hold:

1. $$0 \le B_{4}<T_{4}<B_{2}<T_{2}$$,
2. $$0< B_{5}<T_{5} \le B_{3}<T_{3}<B_{1}<T_{1}$$.

### Proof

We note that in the paper Dósa et al. (2015a) we already introduced the first four safe sets (in the same way), with the same properties. In this paper we need the fifth safe set as well, moreover the claims of the lemma hold also for large values of *s*, thus we need to give the proof of the lemma again. In the calculations we generally use Lemma 2, unless stated otherwise.

1. From $r \le \frac{4s+3}{3s+2}$ it follows that $0 \le 4s + 3 - 3rs - 2r = B_4$. From $r > \frac{4}{3}$ and the definition we have that $0 < (3r-4)(s+1) = D_4 = T_4 - B_4$. From $r < \frac{s+2}{s+1}$ it follows $0 < (s+1-sr) - (r-1) = B_2 - T_4$. By $r>1$ we have that $0 < (s+1)(r-1) = T_2 - B_2$.
2. We observe that for positive *s* the inequality $0 < 6s-5r-4rs+6 = B_5$ is equivalent to $r(s) < \frac{6s+6}{4s+5}$, which holds. Lemma 3.6 states that $T_5-B_5=B_4$, and thus using $B_4 > 0$ from Lemma 4.1 we have $T_5-B_5 > 0$. From $r \ge \frac{8s+7}{6s+5}$ it follows that $0 \le 5r-8s+6rs-7 = (2s-2r-rs+2) - (10s-7r-7rs+9) = B_3 - T_5$. From $r > \frac{3s+2}{2s+2}$ it follows that $0 < 2r + 2rs - 3s - 2 = D_3 = T_3 - B_3$. From $r < \frac{2s+1}{s+1}$ it follows that $0 < (s+1-r)-s(r-1) = B_1 - T_3$. By $r>1$ we have that $0 < (s+1)(r-1) = D_1 = T_1 - B_1$. $\square$

We will need some further properties regarding the safe sets. These properties make the later calculations easier.

### Lemma 5

1. $$D_{1}=D_{2}> \max \left\{ B_2, D_3 \right\} $$,
2. $$ B_2 <1$$ and $$ B_1 <s$$,
3. $$T_{3}-T_{5}\ge B_{2}$$,
4. $$B_{2}\ge B_{3},$$
5. $$T_{2}\ge B_{1}$$,
6. $$D_{3}>B_{4}$$,
7. $$T_{4}+D_{3}>B_{2}$$,
8. $$2D_{1}>s$$,
9. $$T_{4}+D_{1}>1$$,
10. $$T_{4}+T_{2}\ge s$$.

### Proof

We generally use Lemma 2 for the lower or upper bounds on *r*(*s*).

1. $D_{1} = D_{2}$ holds directly by definition. For $D_2 > B_2$ we equivalently have $D_2 - B_2 = (s + 1) (r - 1) -( s+ 1 - sr) = 2sr - 2s - 2 + r > 0$, and hence $r(2s+1) > 2s+2$, which holds. Finally, from $D_2 - D_3 = (rs + r - s-1) - (2r - 3s + 2rs - 2) = -rs - r + 2s +1 > 0$ we get $\frac{2s + 1}{s + 1} > r$, which is true.
2. $$B_2 = s + 1 - sr < 1$$, and $$B_1 = s+1-r < s $$ since $$ 1 <r$$.
3. We have $T_{3}-T_{5}-B_{2}=s(r-1)-(10s-7r-7rs+9)-(s+1-sr)=7r-12s+9rs-10\ge 0$ if and only if $r\ge \frac{12s+10}{9s+7}$. This is trivially true for any $s \le q_{7}$, and true for $s>q_{7}$ by Lemma 1.
4. We have $B_{2}-B_{3} = (s+1-sr)-(2s-2r-rs+2)=2r-s-1 \ge 0$ if and only if $r \ge \frac{s+1}{2}$, which holds.
5. $$T_{2}-B_{1} = r-(s+1-r) = 2r-s-1 \ge 0$$.
6. $D_{3}-B_{4} =(2r-3s+2rs-2)-(4s-2r-3rs+3) = 4r-7s+5rs-5 > 0$ if and only if $r > \frac{7s+5}{5s+4}$.
7. $T_{4}+D_{3}-B_{2}>B_{4}+D_{3}-B_{2}=0$, by Lemmas 3.4 and 4.1.
8. $2D_{1}-s=2\left( s+1\right) \left( r-1\right) - s = 2r-3s+2rs-2 > 0$ holds if $r > \frac{3s+2}{2s+2}$, which is true.
9. $T_{4}+D_{1}-1 = (r-1)+\left( s+1\right) \left( r-1\right) -1 = 2r-s+rs-3 > 0$ if and only if $r > \frac{s+3}{s+2}$.
10. $$T_{4}+T_{2}-s = (r-1)+r-s = 2r-s-1 \ge 0$$ since $$r \ge \frac{s+1}{2}$$. $$\square $$

## Algorithm FinalCases

First the loads are zero. The actual loads of the machines will be denoted as $L_{m}$ ($m=1$ or $m=2$) just before assigning the next job. Thus, for example, if $L_{1}$ denotes the actual load of the first machine, then after assigning a job to this machine, the new load will again be denoted by $L_{1}$.

Here we define a subalgorithm, which works (and will be applied) *only if* the next job can be assigned to a machine whose increased load will be within some safe set. We call the algorithm FinalCases. We will say, for the sake of simplicity, that some step is *executed* if the condition of this step is satisfied and the actual job is assigned at this step. Otherwise we say that the step is only *examined*. In other words, entering some step, it is examined whether the condition of the step is fulfilled or not. If yes, the step is executed. If not, the step is not executed. Moreover, for the sake of simplicity, if some step is not executed, we do not write “else if” in the description of the algorithm; if it turns out that the condition of some step is not satisfied, then the algorithm simply proceeds with examining the next step.
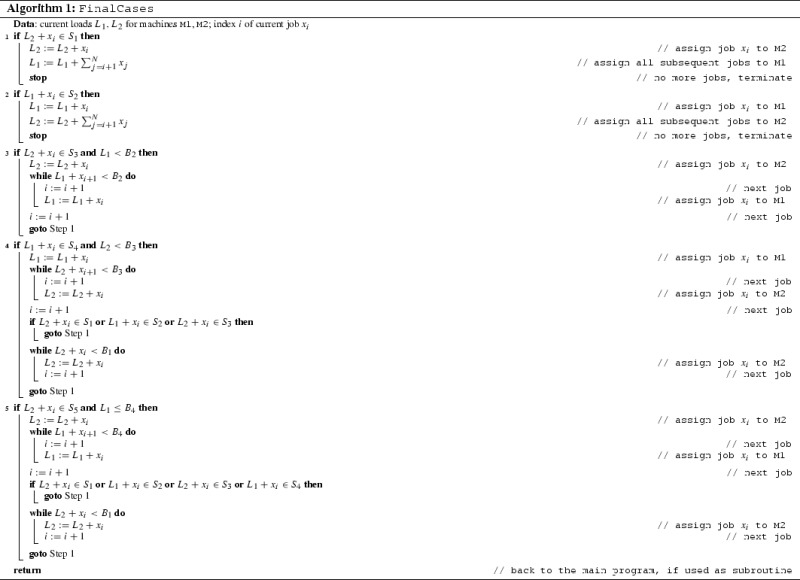


### Theorem 6

Suppose that some of Steps 1 to 5 of Algorithm FinalCases is executed. Then all subsequent jobs are also scheduled by this algorithm, and the competitive ratio is not violated.

### Proof

1. Suppose that Step 1 is executed. Then the load $L_2$ of the fast machine M2 will be not more than $T_{1} = rs$, thus we do not violate the competitive ratio *r* by the fast machine. On the other hand, the final load of the fast machine is at least $B_{1}=s+1-r$, because we assigned job $x_i$ to M2. Applying $\text {SUM} \le s+1$, the final load $L_1$ of the slow machine M1 cannot be more than *r*, since $L_1 = \text {SUM}- L_2 \le (s+1) - (s+1-r) = r$, which means that the competitive ratio is not violated by the slow machine either.
2. Now suppose that Step 2 is executed. The proof is almost the same as for Step 1. The load of M1 does not exceed $T_{2}$, so the competitive ratio is not violated by the slow machine. Moreover the final load of the slow machine is $L_{1}\ge B_{2}=s+1-sr$, thus $L_{2}\le \text {SUM}-L_{1}\le (s+1)-B_{2}=sr=T_{1}$, and we are done.
3. Suppose that Step 3 is executed. After assigning $x_i$ to M2, $B_{3}\le L_{2}\le T_{3}$ holds. Then we possibly assign several jobs to M1. We claim that the increased load of M1 cannot remain below $B_{2}$. Indeed, assume that it stays below $B_2$. Then $B_2 < 1$ from Lemma 5.2, and also $\frac{T_{3}}{s} = r - 1 < 1$ from the rightmost estimation in Lemma 2.1. Hence the makespan would be strictly less than $\text {OPT} = 1$; a contradiction. Thus there must arrive a job that ends the loop, i.e. some job $x_j$ with $L_1 + x_j \ge B_2$. At this point the algorithm goes back to Step 1. We claim that with this job $x_j$ the condition of Step 1 or Step 2 is satisfied, so the algorithm will assign all remaining jobs as explained above, and does not violate the competitive ratio.
  Suppose that the condition of Step 2 is not satisfied, i.e., $L_1 + x_j \notin S_2$. Together with the previously satisfied condition $L_1 + x_j \ge B_2$, we deduce that $L_1 + x_j > T_2$, from which it follows that $x_j > D_2$. We show that in this case the condition of Step 1 is already fulfilled. Indeed, for the lower bound we have $L_2 + x_j > B_3 + D_2 = B_3 + D_1 = B_1$ (where from left to right we applied the condition of Step 3, the definitions of $D_1$ and $D_2$, and Lemma 3.3), while for the upper bound we have $L_2 + x_j \le T_3 + x_j = s(r-1) + x_j = sr - s + x_j = T_1 - s + x_j \le T_1$ (where from left to right we applied the condition of Step 3, the definitions of $T_3$ and $T_1$, and the inequality $x_j \le s$ due to the fact that longer jobs would exceed $\text {OPT} = 1$ even on the fast machine). So we are entering Step 1 or Step 2.
4. Suppose that Step 4 is executed. After assigning $x_i$ to M1, $B_{4}\le L_{1}\le T_{4}$ holds. Then we possibly assign several jobs to M2. We claim that the increased load of M2 cannot remain below $B_{3}$. Indeed, assume that it stays below $B_3$. Then $L_1 \le T_4 = r- 1 < 1$ from Lemma 2.1, moreover $\frac{B_3}{s}< \frac{B_1}{s} < 1$, where we use Lemmas 4.2 and 5.2. Hence the makespan would be strictly less than $\text {OPT} = 1$; a contradiction. Thus there must arrive a job that ends the loop, i.e., some job $x_j$ with $L_2 + x_j \ge B_3$.
  If $L_{2}+x_j$ is in $S_{1}$, or $L_{1}+x_j$ is in $S_{2}$, or $L_{2}+x_j$ is in $S_{3}$, we go back to Step 1. If Step 1 or Step 2 is executed, we are done. Otherwise the condition of Step 3 will be examined. We know that the condition $L_{2} + x_j \in S_{3}$ is fulfilled. Observe that the second condition of Step 3, i.e. $L_{1} \le B_{2}$ also holds, since $L_{1} \le T_{4}$ still holds and we have $T_4 < B_{2}$ from Lemma 4.1. Thus Step 3 is executed, and we are done.
  Now assume that none of the conditions $L_{2}+x_j \in S_{1}$, $L_{1}+x_j \in S_{2}$, or $L_{2}+x_j \in S_{3}$ is satisfied. Let us consider the size of the actual job, $x_j$. Since $L_2 + x_j \ge B_3$ (from the choice of $x_j$), but $L_{2} + x_j$ is not in $S_{3}$, we deduce that $L_2 + x_j > T_3$. Hence together with $L_{2} < B_{3}$ (also from the choice of $x_j$) it follows that $x_j > D_{3}$. Then, using $L_1 \ge B_4$, we get $L_{1} + x_j > B_{4} + D_{3} = B_{2}$ by Lemma 3.4. Since $L_{1} + x_j$ is not in $S_{2}$, we also deduce that $L_{1} + x_j > T_{2}$ holds. On the other hand, the actual load $L_1$ of M1 is at most $T_{4}$. Thus $x_j > T_2 - L_1 \ge T_{2} - T_{4} = 1$, where the equality comes from Lemma 3.2. Suppose that $L_{2}+x_j > T_{1}$. Then $x_j>T_{1}-T_{3}=s$ (by the first part of Lemma 3.2) would follow, which would violate the value of $\text {OPT}$, because even the faster machine M2 can process this job within this makespan. Hence $L_{2}+x_j \le T_{1}$. Together with the fact that $L_2 + x_j \notin S_1$, we have that $L_2 + x_j < B_1$. At this point $x_j$ is assigned to M2 by the algorithm.
  Now several subsequent jobs may be assigned to M2, while the load of M2 remains below $B_{1}$. But, similarly to the previous steps, there must arrive a further job $x_k$ that would exceed $B_1$. Indeed, assume that no such jobs exists. Then $L_1 \le T_4 = r - 1 < 1$ (by Lemma 2.1) and $L_2 \le B_1 < s$ (by Lemma 5.2), so the makespan would stay below $\text {OPT} = 1$; a contradiction. Thus the assignment of jobs to M2 is stopped, and the algorithm goes back to Step 1.
  We claim that one of Step 1 or Step 2 will be executed. If Step 1 is not executed, then $L_2 + x_k \notin S_1$ and $L_2 + x_k > B_1$ from the previous loop. Together, $L_2 + x_k > T_1$. Since $L_2 < B_1$, we obtain $x_k> T_1 - L_2 > T_1 - B_1 = D_{1}$. Then we get $L_{1} + x_k> B_{4}+D_{1} > B_{4} + D_{3}$ by Lemma 5.1, and $B_4 + D_3 = B_{2}$ by Lemma 3.4, hence $L_{1} + x_k > B_{2}$. Assume that Step 2 is not executed either. Then $L_1 + x_k \notin S_2$. Hence $L_1 + x_k > T_2$. From this is follows that $x_k > T_{2} - L_{1} \ge T_{2} - T_{4} = 1$, because $L_1 \le T_4$ is still true and we have $T_{2} - T_{4} = 1$ (from Lemma 3.2). Then there are two jobs, say $x_k$ and $x_j$, which are both bigger than 1, thus both have to be assigned to the fast machine in the optimal schedule. Therefore we have $\text {OPT} > \frac{2}{s}$, and $\frac{2}{s} > 1$ (from $2 > s$), which is a contradiction.
5. Finally, suppose that Step 5 is executed. We assign first the actual job to the machine M2 and then we assign jobs to the machine M1 until $L_1 +x_i < B_4$. Observe that $L_1$ cannot remain below $B_4$. Assume the opposite. Then $L_1 \le B_4< B_2 < 1$ by Lemma 5.2. Moreover, $L_2 \le T_5<B_1 < s$ by Lemma 2.1. Hence the makespan would be strictly less than $\text {OPT} = 1$; a contradiction. Thus there must arrive a job that ends the loop, i.e., some job $x_j$ with $L_1 + x_j \ge B_4$.

If any of the four conditions $L_{1} + x_j \in S_{4}$, or $L_{1} + x_j \in S_{2}$, or $L_{2} + x_j \in S_{3}$, or $L_{2} + x_j \in S_{1}$ is satisfied, we go back to Step 1. Note that at this moment $L_{1}< B_{4} < B_{2}$ and $L_{2} \le T_{5} < B_{3}$ (applying Lemma 4). Hence it follows that some of Step 1 – Step 4 must be executed, and we are done. Therefore, suppose that none of the four conditions is satisfied. Let us consider the size of $x_j$.

Since $L_1 + x_j \ge B_4$ (from the choice of $x_j$), but $L_{1} + x_j$ is not in $S_{4}$, we deduce that $L_1 + x_j > T_4$. Hence together with $L_{1} < B_{4}$ (also from the choice of $x_j$) it follows that $x_j > D_{4}$. Then $L_{2} + x_j > B_{5} + D_{4} = B_{3}$, applying $L_2 \ge B_5$ and Lemma 3.4. Since $L_{2} + x_j$ is not in $S_{3}$, it follows that $L_{2} + x_j > T_{3}$. Together with $L_{2} \le T_{5}$, we get $x_j > T_{3} - T_{5}$. Then $L_{1} + x_j > (B_{2}-T_{3}+T_{5})+(T_{3}-T_{5})=B_{2}$, applying $L_{1} \ge 0 \ge B_{2}-T_{3}+T_{5}$ (Lemma 5.3). On the other hand, we know that $L_{1} + x_j$ is not in $S_{2}$, thus it follows that $L_{1} + x_j > T_{2}$. Consequently, using Lemma 3.2, we get $y>T_{2}-T_{4}=1$.

We know that $L_{2} + x_j$ is not in $S_{1}$. Suppose that $L_{2} + x_j > T_{1}$. Then $x_j> T_{1} - L_2 > T_{1} - T_{3} = s$ would follow, applying Lemma 3.1, and $L_2 \le T_5 < T_3$ by Lemma 4.1; a contradiction. Therefore at this point we assign $x_j$ to machine M2, and the increased load of M2 is strictly bigger than $T_{3}$ and strictly smaller than $B_{1}$.

Now several subsequent jobs may be assigned to M2, while the load of M2 remains below $B_{1}$. There must arrive a job, say $x_k$, such that $L_2 + x_k \ge B_{1}$. Indeed, assume that it stays below $B_1$. Since we know that $L_1 \le T_4$, we conclude similarly to the proof of the previous point that this would lead to a makespan strictly less than $1 = \text {OPT}$; a contradiction.

At this point the algorithm goes back to Step 1. We claim that either Step 1 or Step 2 will be executed. If Step 1 is not executed, then $L_2 + x_k > T_1$, since $L_2 + x_k \ge B_1$. This together with $L_2 < B_1$ implies that $x_k > D_{1}$. Therefore we get $L_{1} + x_k> D_{1} > B_{2}$, by Lemma 5.1. If Step 2 is not executed either, which means that $L_1 + x_k \notin S_2$ and hence $L_1 + x_k > T_2$, then $x_k> T_{2} - L_{1} \ge T_{2} - B_{4} > T_{2} - T_{4} = 1$, where we applied $L_1 < B_4$, $B_4 < T_4$ (by Lemma 4.1), and $T_{2} - T_{4} = 1$ (by Lemma 3.2).

Summarizing our analysis, we have two jobs, $x_j$ and $x_k$, both greater than 1, thus both have to be assigned to the fast machine in the optimal schedule. Therefore we have $\text {OPT} > \frac{2}{s}$, and $\frac{2}{s} > 1$ (from $2 > s$), which is a contradiction. Therefore Step 1 or Step 2 has to be executed and we are done. $\square$

We have seen that Algorithm FinalCases solves the problem (does not violate the competitive ratio) if some step of the algorithm is executed. The problem is that it may happen—although only rarely—that no step can be executed because the condition of no step is satisfied. We must take care about these remaining cases. For this we define another algorithm in the next section.

We say that Algorithm FinalCases is *executable* if the condition of some step is satisfied. Summarizing our previous investigations, if Algorithm FinalCases is executable, then doing so we obtain a schedule which does not violate the competitive ratio.

## Algorithm InitialCases

In order to handle the case where Algorithm FinalCases is not executable, we now give the algorithm InitialCases that calls FinalCases as a subroutine.
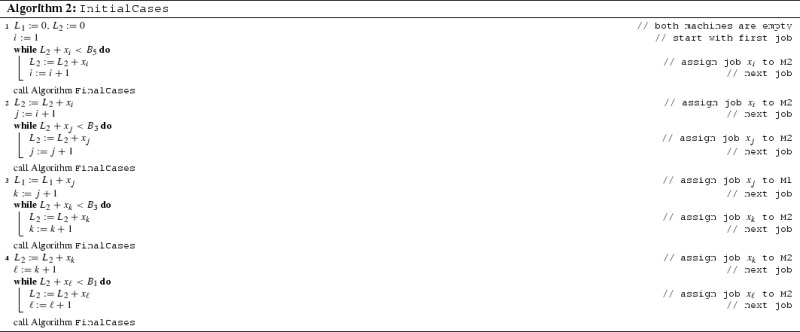


For proving that Algorithm InitialCases is *r*-competitive in the considered interval, we still need one more claim as below.

### Lemma 7

Suppose that machine M1 is empty $(\hbox {i.e.}, L_1 = 0)$, and that the load $L_2$ of machine M2 is at most $B_5$. If *x* is a job whose size satisfies $x \notin S_2$ and $L_2 + x \notin S_1$, then $x \le T_3 - B_5$.

### Proof

Assume that $x > T_3 - B_5$. Since $T_{3}-B_{5} \ge B_{2}$ (by Lemma 5.3), it follows that $x > B_2$. Recall that there is no job assigned to M1 so far. Since $x \notin S_2$, we obtain $x > T_{2}\ge B_{1}$ (where the last estimation was shown in Lemma 5.5). From $x > B_1$ it then follows that $L_2 + x > L_2 + B_1 \ge B_1$. Together with $L_2 + x \notin S_1$, we deduce that $L_2 + x > T_1$. Hence $x> T_1 - L_2 \ge T_1 - B_5 > T_1 - T_3 = s = s \cdot \text {OPT}$ (by Lemma 4.2 and Lemma 3.2). This is a contradiction, since no job can be bigger than $s\cdot \text {OPT}$.$\square$

After this, we state the next theorem.

### Theorem 8

Algorithm InitialCases is *r*-competitive for any $q_6\le s\le \sqrt{3}$.

### Proof

1. If Algorithm FinalCases is called in Step 1 and there all jobs are assigned to machines (in Step 1 and Step 2 of Algorithm FinalCases), then FinalCases terminates and all jobs are within the safe sets, so the competitive ratio of *r* is not exceeded.

At the end of Step 1, let us denote the actual job by $x_i$. It holds that $L_2 + x_i \ge B_5$, and before $x_i$ came, $L_2$ was below $B_5$. Algorithm FinalCases was called at the end of Step 1, but none of the conditions of the five Steps 1–5 in Algorithm FinalCases was actually true (i.e., FinalCases was not executable). In particular, Step 5 of FinalCases was not executed. Since $L_1 = 0$ (machine M1 is empty), and $B_4 > 0$ (from Lemma 4.1), it thus follows that $L_2 + x_i > T_5$. Together with $L_2 < B_5$ it follows from Lemma 3.6 that $x_i > T_5 - B_5 = B_4$. Note that at this point still there is no job assigned to M1. Since $x_i$ is not assigned to M1 (as FinalCases was not executable), in particular, Step 4 of FinalCases is not executable. Since $L_2< B_5 < B_3$ (from Lemma 4.2), it means that $L_1 + x_i \notin S_4$. From $x_i > B_4$ (see above) it then follows that $x_i > T_{4}$.

Suppose that $x_i > B_{3}$ holds (from which we derive a contradiction). Then it follows that $x_i > T_{3}-B_{5}$, because otherwise, if $x_i \le T_3-B_5$, then $T_3 \ge x_i + B_5> x_i + L_2> x_i > B_3$, hence $L_2 + x_i \in S_3$. Since $L_1 = 0 < B_2$ (from Lemma 4.1), it follows that Step 3 of Algorithm FinalCases would be executed; a contradiction. Hence $x_i > T_3 - B_5$. Note that all assumptions of Lemma 7 are satisfied. Hence $x_1 \le T_3 - B_5$; a contradiction. Therefore $x_i \le B_3$.

Thus we conclude from the previous two paragraphs that $T_{4}< x_i < B_{3}$.

Let us investigate how big the actual load of M2 would be, if $x_i$ was assigned to this machine; that is, we want to estimate $L_2 + x_i$. We are going to show that $T_5< L_2 + x_i < B_3$, by excluding all other possibilities. To prove the lower bound, note that since the algorithm terminated the while-loop, we have $L_2 < B_5$ and $L_2 + x_i \ge B_{5}$. As we argued above, we know that $L_2 + x_i \notin S_5$, hence we have $L_2 + x_i > T_5$. To prove the upper bound, we need to exclude two more cases (see also Fig. 2).

- Suppose that $L_2 + x_i \in S_3 = [B_3,T_3]$. Since $L_1 = 0 \le B_2$ (by Lemma 4.1), Step 3 of Algorithm FinalCases would have been executed; a contradiction. Thus $L_2 + x_i \notin [B_3,T_3]$.
- Suppose that $L_2 + x_i > T_3$. Then $x_i> T_3 - L_2 > T_3 - B_5$ (since $L_2 < B_5$, see above). Note that all assumptions of Lemma 7 are satisfied. Hence $x_1 \le T_3 - B_5$; a contradiction. Thus $L_2 + x_i \le T_3$.

Consequently, $$T_5< L_2 + x_i < B_3$$.

2. We enter Step 2. We assign $x_i$ to M2. From the analysis above we know that the load $L_2$ after this assignment is above $T_{5}$ and below $B_{3}$.

Then several jobs may come, and they are assigned to machine M2, while the load $L_2$ of M2 remains below $B_{3}$. This termination point of the while-loop will come for sure: otherwise we would have an empty machine M1, and the total load of all jobs, all on machine M2, would be still below $B_3$. Since $B_3< B_1 <s = s \cdot \text {OPT}$ (by Lemmas 5.2 and 4.2), this contradicts the assumption that the optimum value is $\text {OPT}$.

Let $x_j$ denote the job upon terminating the while-loop. Now we call Algorithm FinalCases with this index *j*. Assume FinalCases is not executable (otherwise we are done). It holds that $L_2 < B_3$ and $L_2 + x_j \ge B_3$. Furthermore, $L_1 = 0 \le B_2$ (by Lemma 4.1), but Step 3 of Algorithm FinalCases was not executed, thus $L_2 + x_j \notin S_3$. Consequently, $L_2 + x_j > T_3$, and thus $x_j > D_3$. By Lemma 5.6 we have $D_3 > B_4$. Since no job is assigned to M1, and Step 4 of FinalCases was not executable, moreover $L_2 \le B_3$, we have that $L_1 + x_j = x_j \notin S_4$. From $x_j> D_3 > B_4$ we deduce $x_j > T_4$.

The assumption of $x_j \ge B_{2}$ will lead to a contradiction as follows. Since Step 2 of FinalCases was not executable, it holds that $L_1 + x_j = x_j \notin S_2$, hence $x_j > T_2$. In Lemma 5.5 we proved that $T_2 > B_1$, hence $x_j > B_1$. Since also Step 1 of FinalCases was not executable, it holds that $L_2 + x_j \notin S_1$. From $x_j > B_1$ we thus deduce that $L_2 + x_j > T_1$. Thus we estimate $x_j> T_1 - L_2 > T_1 - T_3 = s$, where the second estimation uses $L_2< B_3 < T_3$ and the last inequality is due to Lemma 3.2. Hence $x_j > s = s \cdot \text {OPT}$, so job $x_j$ would be too large for an optimum value of $\text {OPT}$.

Summing up, we conclude that $T_{4}< x_j < B_{2}$ holds.

3. In Step 3 we assign $x_j$ to M1, and since this is the only job which has been assigned to $M_{1}$ ever, the load $L_1$ of M1 is between $T_{4}$ and $B_{2}$.

Then again, several jobs may come, and they are assigned to machine M2, while the load $L_2$ of M2 remains below $B_3$. This termination point of the while-loop will come for sure: otherwise we would have machine M1 with a load lower than $B_2 < 1 = \text {OPT}$ by Lemma 5.2, and the load of $L_2$ is below $B_3< B_1 < s = s \cdot \text {OPT}$ by Lemma 5.2. This contradicts the assumption that the optimum value is OPT.

Let $x_k$ denote the job upon terminating the while-loop. Now we call Algorithm FinalCases with this index *k*. Assume that FinalCases is not executable (otherwise we are done). In particular, Step 3 of FinalCases was not executable, and since $L_1 \le B_2$, it follows that $L_2 + x_k \notin S_3$. Taking into account that $L_2 < B_3$ and $L_2 + x_k \ge B_3$, it follows that $L_2 + x_k > T_3$, hence $x_k > D_3$.

From Lemma 5.7 it follows that $x_j + x_k - B_{2}> T_{4} + D_{3} - B_{2} > 0$, thus $x_j + x_k > B_{2}$. Since Step 2 of FinalCases was not executable, it means that $L_1 + x_k = x_j + x_k \notin S_2$, hence $x_j + x_k > T_2$. Since $L_1 = x_j \le B_2$, we have $x_k > D_2 = D_1$ (by the definition of $D_1$ and $D_2$).

Assume that $L_2 + x_k \ge B_1$. Since Step 1 of FinalCases was not executed, it would follow that $L_2 + x_k \ge T_1$. Thus taking into account that $L_2 < B_3$, we obtain the estimation $x_k \ge T_1 - L_2> T_1 - B_3 > T_1 - T_3 = s = s \cdot \text {OPT}$ (by Lemma 4.2 and Lemma 3.2), which contradicts the optimality of value OPT. Thus $L_2 + x_k < B_1$.

4. We start Step 4 with assigning $x_k$ to M2. Then the new load $L_2$ is between $T_3$ and $B_1$.

Then for the last time, several jobs may come, and they are assigned to machine M2, as long as the load $L_2$ of M2 remains below $B_1$. The termination point of the while-loop will come for sure: otherwise we would have a machine M1 with a load lower than $B_2 < 1 = \text {OPT}$ (by Lemma 5.2), and the load of $L_2$ is below $B_1 < s = s \cdot \text {OPT}$ by Lemma 5.8. This contradicts the assumption that the optimum value is OPT.

Let $x_{\ell }$ denote the job upon terminating the while-loop. We will show that now FinalCases *is* executable, thus we are done. Assume the opposite: FinalCases is not executable.

At this point we have $L_{2} < B_{1}$ and $L_{2}+x_{\ell } \ge B_{1}$. Since FinalCases is not executable, in particular, Step 1 of FinalCases was not executable, meaning $L_2 + x_{\ell } \notin S_1$. Hence $L_2 + x_{\ell } > T_1$, thus $x_{\ell } > D_1$.

Using $x_j > T_4$ from Step 2 above, we can estimate $x_j + x_{\ell }> T_{4} + D_{1}> D_1 > B_{2}$ using Lemmas 4.1 and 3.1. Since at this point only $x_j$ is assigned to M1, and Step 2 of FinalCases is not executable, that is $L_1 + x_{\ell } = x_j + x_{\ell } \notin S_2$, it also holds that $x_j + x_{\ell } > T_{2}$.

We summarize: $x_i,x_j > T_{4}$, $x_k, x_{\ell } > D_2 = D_1$, moreover $x_j + x_k > T_{2}$ and $x_j + x_{\ell } > T_{2}$.

Note that $x_k + x_{\ell }> 2 D_{1} > s = s \cdot \text {OPT}$ (by Lemma 5.8). So it follows that $x_k$ and $x_{\ell }$ must be assigned to different machines in any optimum schedule, because even the faster machine M2 cannot handle both jobs within a makespan of OPT.

First, consider an optimum schedule where $x_k$ is assigned to the slower machine M1 and $x_{\ell }$ is assigned to the faster machine M2. Assume that $x_i$ is also assigned to M1. Then we can estimate the load of this machine: $L_1 \ge x_k + x_i> D_1 + T_4 > 1 = \text {OPT}$ (by Lemma 5.9); a contradiction. Hence $x_i$ cannot be assigned to M1. Similarly, if we assume that $x_j$ is assigned to M1, we can deduce the very same estimation. Hence also $x_j$ cannot be assigned to M1. So both $x_i$ and $x_j$ must be assigned to the faster machine M2.

Second, consider an optimum schedule where $x_{\ell }$ is assigned to the slower machine. Then by repeating the same arguments as above, we can deduce that also in this case, both $x_i$ and $x_j$ must be assigned to the faster machine M2.

Thus in any optimal schedule, both $x_i$ and $x_j$ are assigned to the fast machine M2, and one of $x_k$ and $x_{\ell }$ is also assigned to the fast machine. Thus by Lemma 5.10 we get $s \cdot \text {OPT} \ge \min \{ x_i+x_j+x_k,x_i+x_j+x_{\ell })\} =x_i + \min \{ x_j+x_k,x_j+x_{\ell } \} >T_{4}+ \min \{T_{2}, T_2 \} = T_4 + T_2 \ge s = s\cdot \text {OPT}$; a contradiction.

It follows that our assumption was false, i.e., when job $x_{\ell }$ is revealed, FinalCases is executable. This completes the proof. $\square$

## Conclusions

We gave a compound algorithm and showed that its competitive ratio equals the previously known lower bound for any speed $s\in [\frac{5+\sqrt{241}}{12}, \sqrt{3}] \approx [1.7103, 1.7321]$, i.e. on the “wide” interval. Although the considered interval is in fact “not too wide”, we applied new ideas, to be able to get the tight ratio here.

Our idea (as we described it also in the Introduction) in the algorithm design is as follows. Instead of having a universal algorithm, we have two algorithms: one for the “good cases” and another for the problematic cases. If the incoming job is *good* in some sense for us, we assign it with the first algorithm. Otherwise, if the incoming job is *bad*, we assign it by the second algorithm. (Of course, we make only one common schedule, the next job is assigned by the rule of either the first, or the second algorithm, but not both.) The good or bad status of the incoming job depends on its size, and on the actual values of the loads of the machines as well.

If, at any time, a good job arrives, we win against the adversary list, as we are able to finish the schedule by the first algorithm, without violating the prescribed competitive ratio. And it turns out that in any sequence there *must* come a good job. It means that the problematic cases are intermediate cases, and if we can “survive” these problematic cases without making a bad decision (that would lead us to violate the competitive ratio), sooner or later a good case must come.

Except for the narrow interval (which is approximately [1.6934, 1.6963]) where the gap between the upper and lower bounds is very small, the question about the tight value of the competitive ratio for our problem remains open for speeds between $\frac{\sqrt{73}+3}{8} \approx 1.443$ and $\frac{5}{3}$. We think that the applied ideas can be helpful to get the tight ratio (or a ratio which is close to the tight ratio), where the question is actually open.

We also performed computational experiments, which are consistent with our theoretical results. Among other details, these investigations can be found in Dósa et al. (2015b).
